# Effects of Aging on the Structure and Expression of NMDA Receptors of Somatostatin Expressing Neurons in the Mouse Hippocampus

**DOI:** 10.3389/fnagi.2021.782737

**Published:** 2021-12-23

**Authors:** Yaiza Gramuntell, Patrycja Klimczak, Simona Coviello, Marta Perez-Rando, Juan Nacher

**Affiliations:** ^1^Neurobiology Unit, Program in Neurosciences and Institute of Biotechnology and Biomedicine (BIOTECMED), Universitat de València, Burjassot, Spain; ^2^Spanish National Network for Research in Mental Health, Centro de Investigación Biomédica en Red de Salud Mental (CIBERSAM), Madrid, Spain; ^3^Fundación Investigación Hospital Clínico de Valencia, INCLIVA, Valencia, Spain

**Keywords:** hippocampus, interneuron, somatostatin, aging, spine, NMDA – receptor

## Abstract

Changes in the physiology, neurochemistry and structure of neurons, particularly of their dendritic spines, are thought to be crucial players in age-related cognitive decline. One of the most studied brain structures affected by aging is the hippocampus, known to be involved in different essential cognitive processes. While the aging-associated quantitative changes in dendritic spines of hippocampal pyramidal cells have already been studied, the relationship between aging and the structural dynamics of hippocampal interneurons remains relatively unknown. Spines are not a frequent feature in cortical inhibitory neurons, but these postsynaptic structures are abundant in a subpopulation of somatostatin expressing interneurons, particularly in oriens-lacunosum moleculare (O-LM) cells in the hippocampal CA1. Previous studies from our laboratory have shown that the spines of these interneurons are highly plastic and influenced by NMDA receptor manipulation. Thus, in the present study, we have investigated the impact of aging on this interneuronal subpopulation. The analyses were performed in 3−, 9−, and 16-month-old GIN mice, a strain in which somatostatin positive interneurons express GFP. We studied the changes in the density of dendritic spines, *en passant boutons*, and the expression of NMDA receptors (GluN1 and GluN2B) using confocal microscopy and image analysis. We observed a significant decrease in dendritic spine density in 9-month-old animals when compared with 3-month-old animals. We also observed a decrease in the expression of the GluN2B subunit in O-LM cells, but not of that of GluN1, during aging. These results will constitute the basis for more advanced studies of the structure and connectivity of interneurons during aging and their contribution to cognitive decline.

## Introduction

Aging is a natural process related to the gradual loss of physiological, behavioral, and social functions. Therefore, it has an essential impact on the nervous system, and it is considered a risk factor for many neurodegenerative and psychiatric illnesses (Hou et al., 2019). Thus, understanding the neurobiology underlying age-related impairment is essential given the growing elderly population. The structural and functional brain changes observed during aging in the central nervous system are the consequence of cellular and molecular alterations, which are in turn regulated by genetic, epigenetic, lifestyle, and environmental factors (Khan et al., 2017). Interestingly, the brain is not homogeneously affected by aging; specific regions are specially altered. Such is the case of the hippocampus, which is our region of interest in the present study, and appears to be among the most affected.

During aging, the hippocampus shows a decrease in volume, which correlates with a decline in learning and memory (Driscoll et al., 2006). Aging is also associated to the presence of neuroinflammation in this region, along with an up-regulation of pro-inflammatory genes, resulting in oxidative stress (Gavilán et al., 2007; Barrientos et al., 2015). Most studies have found no evidence for the death of pyramidal neurons during aging, both in humans and mice (West et al., 1994). In the case of inhibitory neurons there is still controversy, while some studies have found loss of interneuronal markers in the hippocampus of aged animals (Potier et al., 2006; Stanley et al., 2012), others did not find it (Miettinen et al., 1993). At the cellular level, there are structural alterations in hippocampal pyramidal neurons, such as a reduction in dendritic branching (Markham et al., 2005), and reductions in the density of dendritic spines and synapses (Dickstein et al., 2013). However, little is known about the impact of aging on hippocampal inhibitory circuits. Some studies have described decreased GAD65, GAD67 and specifically, somatostatin mRNA levels in the hippocampus during aging (Vela et al., 2003; Gavilán et al., 2007), but nothing is known on the effects of aging on the structure of hippocampal inhibitory neurons.

The study of interneurons in general, and particularly in the hippocampus, represents a challenge due to the existence of several subpopulations, presenting diverse morphological, neurochemical, physiological, and synaptic characteristics (Booker and Vida, 2018). In the hippocampus, interneurons represent 10–15% of the total neuronal cell population (Pelkey et al., 2017). Particularly, the *oriens-lacunosum moleculare* (O-LM) cells, which are the subpopulation studied in the present work, represent the 4.5% of the total number of CA1 interneurons (Bezaire and Soltesz, 2013). They are characterized by the expression of the neuropeptide somatostatin (SST) (Freund and Buzsáki, 1996; Oliva et al., 2000), and are widely distributed in the different regions of the hippocampus, including CA1 (Köhler and Chan-Palay, 1982; Freund and Buzsáki, 1996). O-LM cells have their soma and main dendritic arbor in the *stratum oriens*, where they receive inputs from pyramidal neurons of the *stratum pyramidale* (Lacaille and Schwartzkroin, 1988; Blasco-Ibáñez and Freund, 1995; Katona et al., 1999). The anatomy of these cells is described in detail in Freund and Buzsáki (1996). Most of these synaptic contacts are established on dendritic spines, a peculiar characteristic of SST-expressing interneurons (Freund and Buzsáki, 1996; Guirado et al., 2014). The O-LM cells reciprocally synapse onto pyramidal and non-pyramidal neurons in the *stratum lacunosum moleculare*, through a dense axonal projection field decorated with abundant *en passant boutons* (EPB) (Sik et al., 1995; Müller and Remy, 2014). This anatomical arrangement of the O-LM cells allows them to function in a prototypical feedback inhibitory circuit (Leão et al., 2012), in which they mediate theta oscillations (Katona et al., 2016).

Because of their roles as postsynaptic and presynaptic elements, dendritic spines and synaptic boutons respectively are proper proxies for neuronal input and output; therefore, increases in their density have been correlated to increases in neuronal activity (Engert and Bonhoeffer, 1999; Becker et al., 2008). Our laboratory has previously demonstrated the structural remodeling of the dendritic arbor and dendritic spines of O-LM cells by chronic stress (Gilabert-Juan et al., 2017) and by the depletion of the polysialylated form of the neural cell adhesion molecule (PSA-NCAM), a plasticity-related molecule (Guirado et al., 2014). We also showed that the manipulation of NMDA receptors affected both the density of dendritic spines and EPB (Pérez-Rando et al., 2017a,b).

Apart from these molecular and structural changes, functional impairments are also observed during aging, such as alterations in long-term potentiation (LTP) and depression (LTD) (Lister and Barnes, 2009). Some of the most extensively studied molecules involved in these physiological mechanisms, which underlie basic cognitive processes, are the *N*-methyl-d-aspartate receptors (NMDARs). These are ionotropic glutamate receptors, which are highly expressed in the neocortex and the hippocampus, both in pyramidal and inhibitory neurons, including the O-LM cells (Alvarez et al., 2007; Oren et al., 2009; Pérez-Rando et al., 2017b). The NMDARs are assembled as tetramers composed of 4 subunits: two obligatory GluN1 subunits, along with 2 GluN2 or GluN3 subunits. There are 6 subtypes of non-obligatory subunits: 4 GluN2 (GluN2A–GluN2D) and 2 GluN3 (GluN3A and GluN3B) (Hansen et al., 2017). Alterations in NMDAR complex expression and physiology have been described during aging, especially in the hippocampus (Clayton et al., 2002; Boric et al., 2008; Foster et al., 2017). The GluN2B subunit is more affected by aging than the other GluN2 subunits, showing more significant decreases in mRNA and protein expression in aged animals (Magnusson et al., 2002). The effects of aging on hippocampal GluN1 expression have rendered conflicting results, with significant declines described in some studies, but not in others (Magnusson et al., 2005; Das and Magnusson, 2011). However, the majority of these studies have been performed using western blot or RT-PCR, which does not allow the discrimination of the expression in different hippocampal regions, layers or neuronal populations. SST-expressing interneurons are a very interesting subject to study during aging in this regard, because we know that they express NMDA receptors (Pérez-Rando et al., 2017b) in young adult animals and we have also evidence that these receptors regulate structure and connectivity of the SST-expressing interneurons (Pérez-Rando et al., 2017a,b).

Sex is a very important factor to take into account when studying the nervous system and its pathologies; the study of the female brain in preclinical research is of paramount importance, but studies using both sexes have started to appear published only recently (Shansky and Murphy, 2021). Specifically in the hippocampus, there are sex differences in the dendritic spine density of pyramidal neurons (Shors et al., 2001). These differences are not restricted to excitatory neurons; a recent study from our laboratory has shown changes in the density of dendritic spines and EPB in the O-LM cells during the estrous cycle (Pérez-Rando et al., 2021). The expression of hippocampal NMDARs also appears to be affected by sex and levels of estrogens: Male rats express higher levels of NMDARs in the hippocampus than females, but only when females are in the estrus phase (Brandt et al., 2020). All these studies have been performed in adult-young animals. However, to our knowledge there are still no studies exploring the differential effects of sex on interneuronal morphology or NMDARs expression during aging.

The present study aimed to understand the impact of age on the structure of O-LM cells in the CA1 hippocampal region of male and female mice. We have also studied the expression of different subunits of NMDARs (GluN1 and GluN2B) in this interneuronal subpopulation.

## Materials and Methods

### Animals

Thirty transgenic mice [GIN (GFP-expressing Inhibitory Neurons), Tg(GadGFP)45704Swn] (Jackson Laboratories, Bar Harbor, Maine, United States) were used in this study. They constitutively express the green fluorescent protein (GFP) in a subpopulation of SST-expressing interneurons (Oliva et al., 2000). Mice were bred and maintained in our animal facility and were divided into 3 age groups (3 months, 9 months and 16 months-old). All groups contained 5 males and 5 females. Animals were maintained under controlled conditions of temperature (25°C), humidity (50%), with food and water *ad libitum* and on a standard light/dark cycle (12 h cycle).

All animal experimentation was conducted in accordance with the Directive 2010/63/EU of the European Parliament and of the Council of 22 September 2010 on the protection of animals used for scientific purposes and was approved by the Committee on Bioethics of the Universitat de València. Every effort was made to minimize the number of animals used and their suffering.

### Histological Procedures

When they reached 3, 9, or 16 months-old, mice were deeply anesthetized with pentobarbital and perfused transcardially, first for 1 min with saline (NaCl 0.9%) and then for 30 min with 4% paraformaldehyde in sodium phosphate buffer 0.1 M, pH 7.4 (PB). The left hemisphere was cut in 100 μm-thick coronal sections with a vibratome (Leica VT 1000E, Leica, Nussloch, Germany) to analyze dendritic spine and EPB density on GFP expressing interneurons. The right hemisphere was cut in 50 μm-thick coronal sections for the study of the expression of NMDARs.

### Analysis of the Density of Dendritic Spines and Axonal *En Passant Boutons*

One subseries of sections from each animal was processed “free-floating” for GFP immunohistochemistry. Sections were first washed three times with phosphate buffered saline (PBS) for 10 min per washing. After that, sections were incubated for 1 min in an antigen unmasking solution (0.01 M citrate buffer, pH 6) at 100°C. Then, sections were washed as described above. To block non-specific unions, sections were treated for 1 h with 10% normal donkey serum (NDS) (Jackson ImmunoResearch Laboratories West Grove, PA, United States) in PBS with 0.2% Triton-X100 (Sigma–Aldrich, St. Louis, MO, United States). Sections were washed 3 times in PBS and were incubated for 48 h at 4°C with primary antibody (chicken anti-GFP IgY, Abcam, 1:2000) diluted in PBS 0.2% Triton-X100. After washing, sections were incubated for 2 h at room temperature with a fluorescent secondary antibody (donkey anti-chicken CF488A, Biotum, 1:800) diluted in PBS 0.2% Triton-X100. Sections were then rinsed with PB 0.1 M, mounted on slides and coverslipped using Dako fluorescent mounting medium (Agilent, United States).

For the study of GFP + interneurons, we used a laser scanning confocal microscope (Leica, SPE, Leica Microsystems, Wetzlar, Germany), obtaining 3D stacks of confocal images with 0.38 μm Z-step size. In order to be analyzed, we selected dendrites from GFP interneurons with their soma *stratum oriens* and axons in *stratum lacunosum-moleculare* of the CA1 region.

For the spine density analysis, a 63× objective with a 3.5× digital zoom was used. Dendrites had to fulfill the following features: (1) they should measure at least 150 or 200 μm from the soma; and (2) no other dendrites should be found crossing their trajectory. Furthermore, dendritic spines were defined as clear protrusions emerging from the dendritic shaft. According to these features, we randomly selected six isolated GFP-expressing interneurons per animal, in which the spines were quantified in 3 successive segments of 50 μm up to a total length of 150 μm, using ImageJ (FIJI) (Schindelin et al., 2012). Overall spine density values or densities per segment were expressed as the number of spines/150 μm or spines/50 μm, respectively.

For the EPB density analysis we used a 63× objective with a 2.5× digital zoom. EPB were considered when they fulfilled the following features: (1) they should be at least two times brighter than the axonal backbone; (2) they should be two times wider than the axonal backbone; and (3) they should not have any crossings from other axons nearby. Then, after selecting six random axonal segments per animal that measured at least 10 μm, we used ImageJ (FIJI) (Schindelin et al., 2012) to quantify the number of EPB. The EPB density values were expressed as the number of EPB/μm.

### Analysis of GluN1 and GluN2B Expression

The immunohistochemical protocol employed was similar to that described above for GFP immunohistochemistry. For every subunit of NMDAR (GluN1 and GluN2B) we used different subseries of sections. In order to study the GluN1 expression, sections were incubated with rabbit anti-GluN1 (Alomone, 1:400) or GluN2B rabbit anti-GluN2B (Alomone, 1:4000) together with chicken anti-GFP IgY (Abcam, 1:500) primary antibodies for 48 h at 4°C. After washing, sections were incubated for 2 h at room temperature with donkey anti-rabbit (Biotium, A555, 1:800) and donkey anti-chicken, (Biotium, CF488A, 1:800) secondary antibodies. Sections were then rinsed with PB 0.1 M, mounted on slides and coverslipped using Dako fluorescent mounting medium (Agilent, United States).

We used a laser scanning confocal microscope (Leica, SPE, Leica Microsystems, Wetzlar, Germany), obtaining 3D stacks of confocal images with a 63× objective, 3.5× digital zoom and 0.38 μm Z-step size for the study of GluN1 and GluN2B expression on the somata of GFP expressing interneurons. Ten isolated GFP-expressing somata per animal were selected randomly in the CA1 *stratum oriens*. Controls were performed omitting the anti-GluN1 or anti-GluN2B antibody, as well as incubating with these antibodies previously pre-absorbed overnight with an excess of its immunogenic peptide (GluN1 blocking peptide, Alomone, Jerusalem, Israel) or (GluN2B blocking peptide, Alomone, Jerusalem, Israel), respectively. No immunolabeling was observed in these controls.

Images were processed using Fiji software (Schindelin et al., 2012) as follows: the background was subtracted with a rolling value of 50, converted to 8-bit deep images and binarized using a determined threshold value. This value depended on the marker, but was kept the same for all images with the same marker. Finally, the software counted the number of puncta per cell and the percentage of area covered with these puncta. For the image analysis of GluN1, we outlined manually the profile of the cell somata and used a series of custom-made macros in Fiji, as previously described (Guirado et al., 2018). The original outline was expanded 0.5 μm from the cell body surface, obtaining two regions of interest (ROIs), the cell somata (the original outline) and the cell periphery (including the plasma membrane) (the area between both outlines). Then, ROIs were converted to 8-bit deep images and binarized using a determined size particle (larger than 0.04 μm) and threshold value. Finally, the software calculated the density of puncta and the percentage of area covered with them, both in cell somata and in the periphery. For GluN2B, we followed the same procedure, but in the 16 months-old animals, since puncta were frequently clustered into larger structures, the macro was adjusted and the threshold parameters were modified, in order to determine the percentage of area covered with these clusters of the receptor.

### Statistics

The statistical analysis was based on the indications of Diester et al. (2019). We first analyzed pooled data from both sexes without considering the sex factor, and then data were segregated by sex and analyzed separately. The sex factor was not analyzed since the differences between sexes were not an objective of our study. After checking the normality and homoscedasticity of the data, one way ANOVA or Welch ANOVA tests were used to analyze the density of EPB and the density and percentage of area covered with GluN1 and GluN2B in GFP + somata during the aging process. A significant one-way ANOVA or Welch ANOVA was followed with the correspondent Tukey or Dunnett *post hoc* tests, respectively. Mean ± SEM was used in all cases. For the analysis of dendritic spine density, different generalized linear mixed models (GLMM) of the Poisson family were performed. To evaluate which variables were significant in the model, we determined the Bayesian Information Criteria (BIC). Using the non-interaction models obtained by the BIC, we determined the effect of age and segment in the density of dendritic spines performing a Wald test. A significant Wald test was followed by Tukey’s *post hoc* test. In every case α was set to 0.05. All the analyses were performed using the R studio 1.3.1093 software. Graphs were generated with Graphpad Prism 8.4.3 software.

## Results

### Dendritic Spine Density Analysis

**FIGURE 1:**
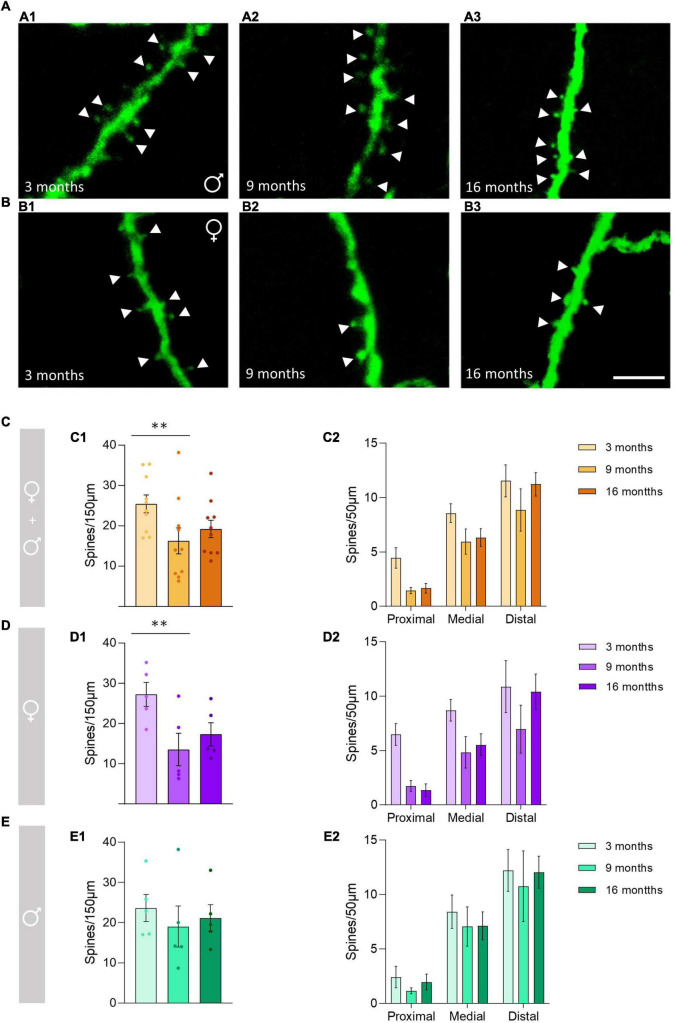
Changes in the dendritic spine density of the *stratum oriens* O-LM cells during aging. **(A)** Segments of dendrites bearing spines (arrowheads) from 3-month-old **(A1)**, 9-month-old **(A2)**, and 16-month-old **(A3)** male mice. **(B)** Segments of dendrites bearing spines (arrowheads) from 3-month-old **(B1)**, 9-month-old **(B2)**, and 16-month-old **(B3)** female mice. **(C–E)** Graphs showing the density of the dendritic spines in pooled females and males, and animals segregated by sex in the total length of the dendrite analyzed (150 μm) **(C1–E1)** and in the proximal, medial and distal segment relative to the soma **(C2–E2)** (all graphs represent mean ± SEM., **p-value < 0.01). Scale bar: 5 μm.

To determine the effect of aging on the density of dendritic spines of O-LM cells (Figures 1A,B) in the whole (0–150 μm) dendritic segments, pooled males and females were analyzed without considering the segment factor (proximal, medial and distal). Since non-interaction models were chosen (see “Statistics” section), the effect of age in each segment (age × segment) could not be analyzed and compared; nevertheless, the results are shown in graphs Figures 1C2–E2. When analyzing all animals together, we observed a significant decrease in the density of dendritic spines between 3− and 9-month-old animals (^**^p = 0.006), but no significant differences were observed between 3− and 16-month-old animals (*p* = 0.22) or between 9− and 16-month-old animals (*p* = 0.32) (Figure 1C1). When females were analyzed separately, we found a significant decrease in the density of dendritic spines between 3− and 9-month-old animals (^**^p = 0.003), but no differences were observed between 3− and 16-month-old animals (*p* = 0.14) or between 9− and 16-month-old animals (*p* = 0.37) (Figure 1D1). When males were analyzed separately no significant effect of age was found (*p* = 0.44; Figure 1E1). The statistics of the correspondent test are included in the Table 1.

**TABLE 1:** Summary of the results of the dendritic spine density and *en passant boutons.*

	Dendritic spines (150 m)								En passant boutons							
	Test	DF	F	*P*-value	*Post hoc*	3–9 months	9–16 months	3–16 months	Test	DF	F	*P*-value	*Post hoc*	3–9 months	9–16 months	3–16 months
Pooled females and males	Wald	2	4.77	0.008**	Tukey	0.006**	0.32	0.22	One way ANOVA	2	1.62	0.22	N/A	N/A	N/A	N/A
Females	Wald	2	5.34	0.005**	Tukey	0.003**	0.37	0.14	One way ANOVA	2	0.62	0.56	N/A	N/A	N/A	N/A
Males	Wald	2	0.82	0.44	N/A	N/A	N/A	N/A	One way ANOVA	2	2.06	0.17	N/A	N/A	N/A	N/A

**p < 0.01.

*When the test was not significant, post hoc comparisons were not applicable (N/A).*

### *En Passant Bouton* Density Analysis

**FIGURE 2:**
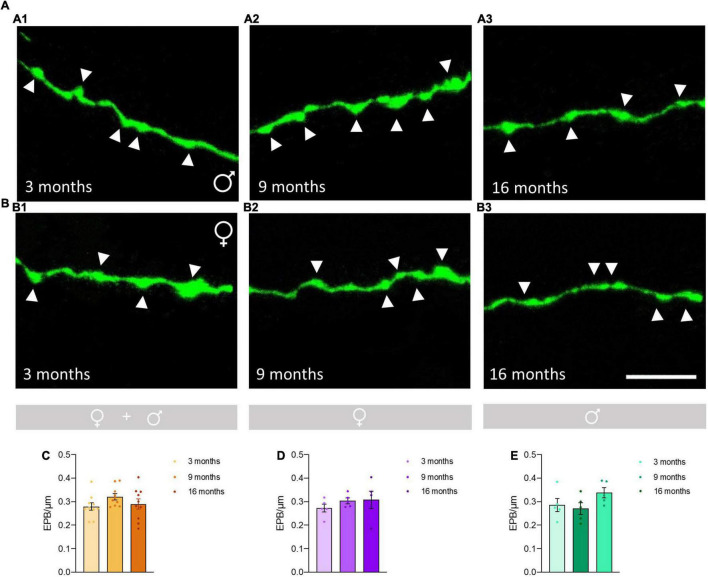
Analysis of the effects of aging on the density of *en passant boutons* (EPB) in axons of O-LM cells in the *stratum lacunosum-moleculare*. **(A)** Axonal segments showing EPB (arrowheads) from 3-month-old **(A1)**, 9-month-old **(A2)**, and 16-month-old **(A3)** male mice. **(B)** Axonal segments showing EPB (arrowheads) from 3-month-old **(B1)**, 9-month-old **(B2)**, and 16-month-old **(B3)** female mice. **(C–E)** Graphs showing the density of EPB in animals segregated by sex **(C),** pooled females **(D)** and males (**E**) (all graphs represent mean ± SEM). Scale bar: 5 μm.

No significant differences in the density of *EPB* from O-LM cells were observed between the different age groups (Figures 2A,B), neither when considering females and males together (*p* = 0.22; Figure 2C) nor when females (*p* = 0.56; Figure 2D) and males (*p* = 0.17; Figure 2E) were analyzed separately. The statistics of the correspondent test are included in the Table 1.

### Analysis of GluN1 Immunoreactive Structures in Oriens-Lacunosum Moleculare Cells

**FIGURE 3:**
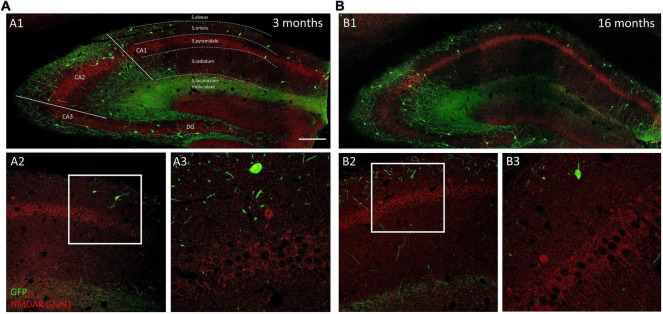
Distribution of GluN1 immunoreactivity in young and aged GIN mice hippocampus. **(A1,B1)** Panoramic confocal planes showing the distribution of O-LM cells (green) and GluN1 immunoreactivity (red) in the hippocampus of 3-month-old **(A1)** and 16-month-old **(B1)** mice. Different regions and strata are indicated with dotted lines. **(A2,B2)** High magnification view from the different CA1 *strata* in 3-month-old **(A2)** and 16-month-old **(B2)** mice. **(A3,B3)** Enlarged view of the squared regions in panels **(A2,B2)**, showing double immunofluorescence for GFP/GluN1, in *strata oriens*, and *pyramidale.* Note the homogenous distribution of GluN1 immunoreactive puncta in pyramidal neurons in both 3-month-old **(A3)** and 16-month-old **(B3)** mice. Scale bar: 150 μm for panels **(A1,B1)**, 67 μm for panels **(A2,B2)**, and 21 μm for panels **(A3,B3)**.

**FIGURE 4:**
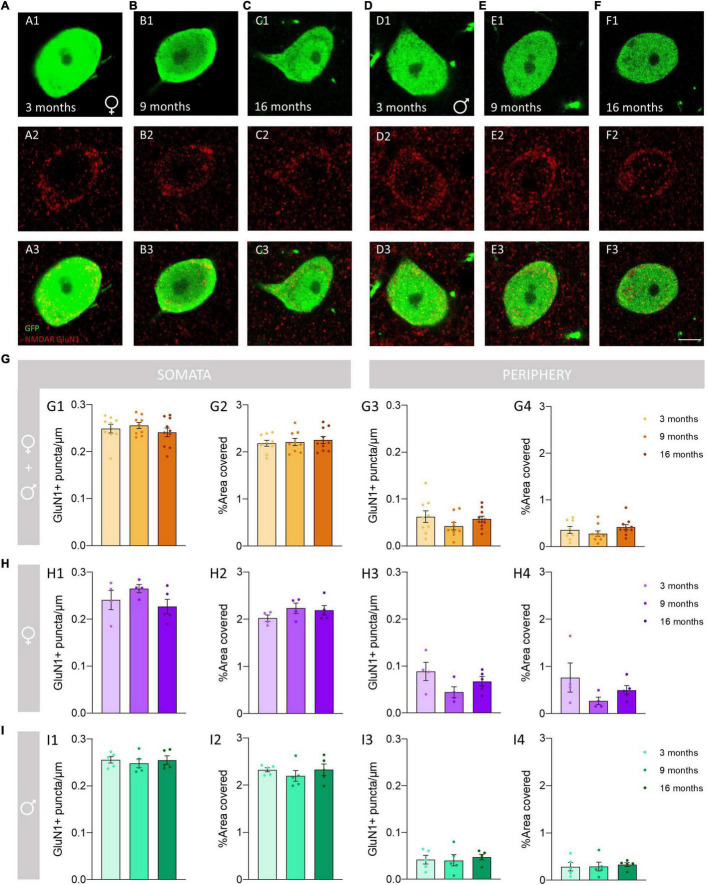
Analysis of the density and percentage of area covered with GluN1 immunoreactive puncta in the somata and the periphery of O-LM cells during aging. **(A–F)** Double GFP/GluN1 immunohistochemistry in 3-month-old **(A)**, 9-month-old **(B)** and 16-month-old **(C)** female mice, and in 3-month-old **(D)**, 9-month-old **(E)** and 16-month-old **(F)** male mice. **(G–I)** Graphs showing the density and percentage of area covered with GluN1 immunoreactive puncta in the somata **(G1,G2–I2)** and in its periphery **(G3,G4–I4)** in animals segregated by sex **(G1–4)**, pooled females **(H1–4)** and males **(I1–4)** (all graphs represent mean ± SEM). Scale bar: 5 μm.

We analyzed the density of GluN1 immunoreactive puncta and the area covered by these structures in the somata and the periphery (see methods) of O-LM cells (Figures 3A,B). The morphology and size of puncta expressing this subunit was similar in 3−, 9− and 16-month-old mice (Figures 4A–F). The analysis of the density of GluN1 immunoreactive puncta in the somata of O-LM cells did not show significant differences due to aging when both sexes were pooled together (*p* = 0.51; Figure 4G1), in females (*p* = 0.26; Figure 4H1) or in males (*p* = 0.78; Figure 4I1). Similar negative results were found when we analyzed the percentage of area of the somata covered with GluN1 + puncta: pooled females and males (*p* = 0.79; Figure 4G2), females (*p* = 0.32; Figure 4H2) and males (*p* = 0.58; Figure 4I2). In the cell periphery the density of GluN1 + puncta also did not show changes during aging: pooled females and males (*p* = 0.30; Figure 4G3), females (*p* = 0.15; Figure 4H3) and males (*p* = 0.86; Figure 4I3). Similarly, we did not detect significant differences in the percentage of area covered with GluN1 + puncta in the periphery of the cells: pooled males and females (*p* = 0.32; Figure 4G4), females (*p* = 0.24; Figure 4H4) and males (*p* = 0.91; Figure 4I4). The statistics of the correspondent test are included in the Table 2.

**TABLE 2:** Summary of the results of the expression of NMDAR.

Density	Test	DF	F	*P*-value	*Post hoc*	3–9 months	9–16 months	3–16 months
	**Pooled females and males**							

NMDAR GluN1 cytoplasm	One way ANOVA	2	0.70	0.51	N/A	N/A	N/A	N/A
NMDAR GluN1 membrane	One way ANOVA	2	1.28	0.30	N/A	N/A	N/A	N/A
NMDAR GluN2B cytoplasm	Welch ANOVA	2	22.59	<0.0001****	Dunnet	0.84	0.040*	<0.0001****
NMDAR GluN2B membrane	One way ANOVA	2	3.93	0.033*	Tukey	0.88	0.10	0.038*
**Percentage of area covered**								
NMDAR GluN1 cytoplasm	One way ANOVA	2	0.24	0.79	N/A	N/A	N/A	N/A
NMDAR GluN1 membrane	One way ANOVA	2	1.21	0.32	N/A	N/A	N/A	N/A
NMDAR GluN2B cytoplasm	Welch ANOVA	2	867.00	<0.0001****	Dunnet	0.55	<0.0001****	<0.0001****
NMDAR GluN2B membrane	One way ANOVA	2	0.96	0.40	N/A	N/A	N/A	N/A

	**Females**							

NMDAR GluN1 cytoplasm	One way ANOVA	2	1.53	0.26	N/A	N/A	N/A	N/A
NMDAR GluN1 membrane	One way ANOVA	2	2.34	0.15	N/A	N/A	N/A	N/A
NMDAR GluN2B cytoplasm	One way ANOVA	2	9.72	0.004**	Tukey	0.57	0.031*	0.003**
NMDAR GluN2B membrane	One way ANOVA	2	0.97	0.41	N/A	N/A	N/A	N/A
**Percentage of area covered**								
NMDAR GluN1 cytoplasm	One way ANOVA	2	1.27	0.32	N/A	N/A	N/A	N/A
NMDAR GluN1 membrane	One way ANOVA	2	1.69	0.24	N/A	N/A	N/A	N/A
NMDAR GluN2B cytoplasm	One way ANOVA	2	570.90	<0.0001****	Tukey	0.84	<0.0001****	<0.0001****
NMDAR GluN2B membrane	Welch ANOVA	2	0.46	0.26	N/A	N/A	N/A	N/A

	**Males**							

NMDAR GluN1 cytoplasm	One way ANOVA	2	0.26	0.78	N/A	N/A	N/A	N/A
NMDAR GluN1 membrane	One way ANOVA	2	0.15	0.86	N/A	N/A	N/A	N/A
NMDAR GluN2B cytoplasm	One way ANOVA	2	3.11	0.09	N/A	N/A	N/A	N/A
NMDAR GluN2B membrane	One way ANOVA	2	5.86	0.018*	Tukey	0.24	0.21	0.015*
**Percentage of area covered**								
NMDAR GluN1 cytoplasm	One way ANOVA	2	0.57	0.58	N/A	N/A	N/A	N/A
NMDAR GluN1 membrane	One way ANOVA	2	0.09	0.91	N/A	N/A	N/A	N/A
NMDAR GluN2B cytoplasm	One way ANOVA	2	1,268.00	<0.0001****	Tukey	0.97	<0.0001****	<0.0001****
NMDAR GluN2B membrane	One way ANOVA	2	0.87	0.45	N/A	N/A	N/A	N/A

p < 0.05*; p < 0.01**; p < 0.0001****.

*When the test was not significant, post hoc comparisons were not applicable (N/A).*

### Analysis of GluN2B Immunoreactive Structures in Oriens-Lacunosum Moleculare Cells

**FIGURE 5:**
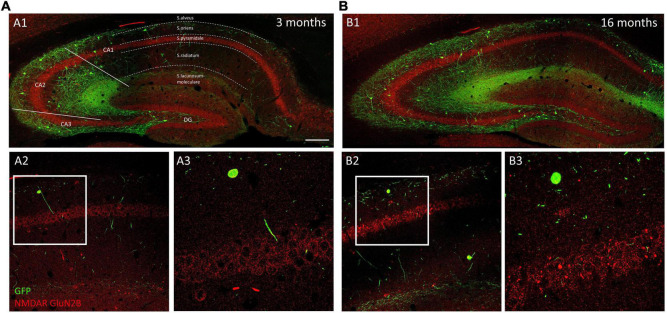
Distribution of GluN2B immunoreactivity in young and aged GIN mice hippocampus. Panoramic confocal plane showing the distribution of O-LM cells (green) and GluN2B immunoreactivity (red) in the hippocampus of 3-month-old **(A1)** and 16-month-old **(B1)** mice. Different regions and strata are indicated with dotted lines. **(A2,B2)** High magnification view from the different CA1 *strata* in 3-month-old **(A2)** and 16-month-old **(B2)** mice. **(A3,B3)** Enlarged view of the squared regions in panels **(A2,B2)**, showing double immunofluorescence for GFP/GluN2B, in *strata oriens*, and *pyramidale.* Note the presence of GluN2B + clusters in pyramidal neurons in 16-month-old **(B3)**, but not in 3-month-old **(A3)** mice. Scale bar: 150 μm for panels **(A1,B1**), 67 μm for panels **(A2,B2)**, and 21 μm for panels **(A3,B3)**.

**FIGURE 6:**
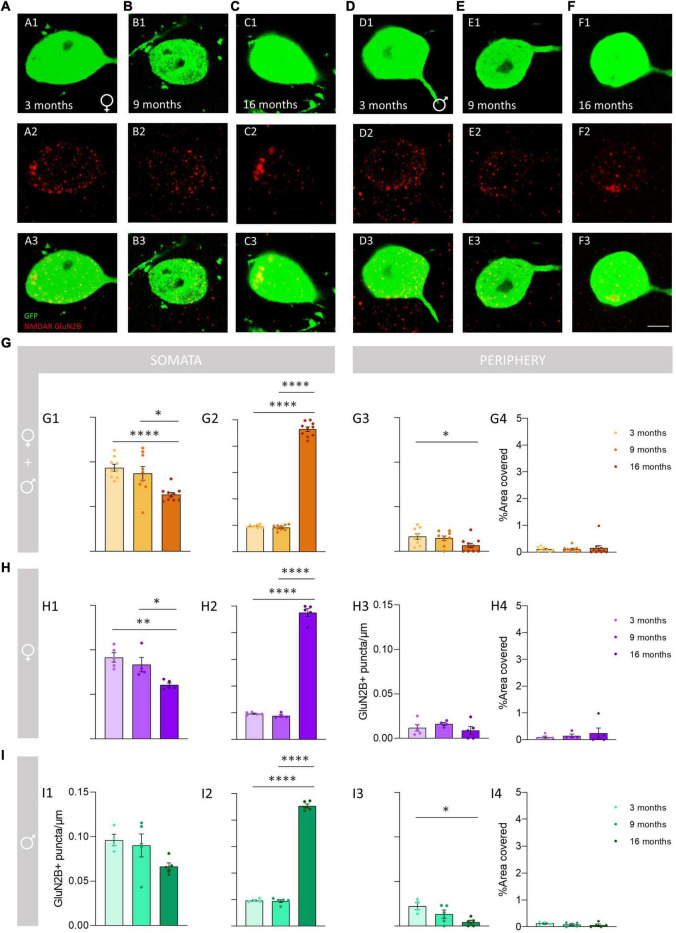
Analysis of the density and percentage of area covered with GluN2B immunoreactive puncta in the somata and in the periphery of O-LM cells during aging. **(A–F)** Double GFP/GluN2B immunohistochemistry in 3-month-old **(A)**, 9-month-old **(B)**, 16-month-old **(C)** female mice and in 3-month-old **(D)**, 9-month-old **(E)**, 16-month-old **(F)** male mice. In panels **(C2,F2)**, a detailed view of the GluN2B clustering in aged mice can be observed. **(G–I)** Graphs showing the density and percentage of area covered with GluN2B immunoreactive puncta in the somata **(G1,G2–I2)** and in its periphery **(G3,G4–I4)** in animals segregated by sex **(G1–4)**, pooled females **(H1–4)** and males **(I1–4)** (all graphs represent mean ± SEM., *p-value <0.05, **p-value <0.01, ****p-value <0.0001). Scale bar: 5 μm.

We analyzed the density of GluN2B immunoreactive puncta and the area covered by these structures in the somata and the periphery of O-LM cells (Figures 5A,B). The morphology and size of the puncta expressing this subunit was similar to that described for GluN1 + puncta in 3− and 9-month-old mice, but in 16-month-old mice we frequently observed the presence of larger structures composed of clustered GluN2B + puncta (Figures 6A–F). The analysis of the density of GluN2B immunoreactive puncta in the soma showed a significant decrease between 3− and 16-month-old animals when both sexes were pooled together (^****^p < 0.0001; Figure 6G1) and in females (^**^p = 0.004; Figure 6H1). Additionally, we also observed a significant decrease between 9− and 16-month-old animals in pooled females and males (*p = 0.040; Figure 6G1), and in females (*p = 0.031; Figure 6H1). However, no significant differences were found between 3− and 9-month-old mice in both sexes together (*p* = 0.84; Figure 6G1) or in females (*p* = 0.57; Figure 6H1). Males did not show significant differences (*p* = 0.09; Figure 6I1). By contrast, the percentage of area covered by GluN2B + puncta in the somata of O-LM cells showed a significant increase when females and males were pooled together and in females and males separately when comparing 3− and 16− (^****^p < 0.0001) and 9− and 16-month-old mice (^****^p < 0.0001) (Figures 6G2–I2). However, no significant differences were found between 3− and 9-month-old individuals in pooled female and male (*p* = 0.55; Figure 6G2), female (*p* = 0.84; Figure 6H2) and male (*p* = 0.97; Figure 6I2) mice. In the cell periphery, the density of GluN2B + puncta showed a significant decrease in females and males pooled together (*p = 0.038; Figure 6G3) and in males (*p = 0.015; Figure 6I3) between 3− and 16-month-old mice. No significant differences were found in pooled females and males an in males between 3− and 9− (*p* = 0.88, Figure 6G3; *p* = 0.24, Figure 6I3) or 9− and 16-month-old mice (*p* = 0.10, Figure 6G3; *p* = 0.21, Figure 6I3). We did not detect significant differences in females (*p* = 0.41; 6H3). Similarly, non-significant differences were observed in the percentage of area in the periphery covered with GluN2B + immunoreactivity: females and males pooled together (*p* = 0.40; Figure 6G4), females (*p* = 0.26; Figure 6H4), and males (*p* = 0.45; Figure 6I4). The statistics of the correspondent test are included in the Table 2.

## Discussion

In the present study, we demonstrate age-dependent alterations in the structure of SST-expressing interneurons in the CA1 *stratum oriens*. There was a significant decrease in the density of the dendritic spines between 3− and 9-month-old females. However, we did not observe such changes in males. We also studied the density of the *en passant boutons* (EPB) of these interneurons in the *stratum lacunosum moleculare*, where no changes were found related to aging. We have focused our study in the *stratum oriens* of the CA1 region. It should be noted that although there is a small proportion of the GFP + cells in this *stratum* that project to the *striatum*, most of them are O-LM cells, which project to *stratum lacunosum moleculare* (Oliva et al., 2000; Pérez-Rando et al., 2017b). The O-LM cells are an interneuronal subpopulation characterized by the presence of abundant spines in their dendritic tree (Freund and Buzsáki, 1996) and numerous EPB in their axonal projection field in the *stratum lacunosum moleculare* (Müller and Remy, 2014). The spines and EPB of O-LM cells have been studied during aging previously, using *in vivo* two-photon imaging in GIN mice, but only from 4 to 11 months of age. The density of EPB in the *stratum lacunosum moleculare* remained constant, similar to what we have observed in fixed tissue. However, in contrast with our results, the spine density of O-LM interneurons increased by 12% from 4 to 11 months (Schmid et al., 2016). However, the big differences in methodology between the 2 experiments have to be taken into account. Ablation of neocortex covering the hippocampus, type of imaging and the age of the animals compared. It is important to note that both sexes were analyzed together in this experiment, without control over the estrous cycle, which can influence spine density in O-LM interneurons (Pérez-Rando et al., 2021). Moreover, this study examined dendritic spines in behaving animals and, consequently, spine density can be influenced by this behavior, possibly through the activation NMDA receptors. To our knowledge the present study is the first to analyze the structure of SST-expressing neurons in intact animals and to include aged animals from both sexes. Age-related structural changes have been also studied in other subpopulations of interneurons, particularly analyzing their dendritic arborization. In the visual cortex, the dendritic arbors of interneurons (in general) were simplified in aged mice (Eavri et al., 2018). However, in other specific interneuronal types, such as the vasoactive intestinal peptide/calretinin-expressing cells in the CA1 region of the hippocampus, no differences were found in dendritic length or number of intersections between young and old mice (Francavilla et al., 2020). However, it has to be noted that we have observed changes in the dendritic spine density only in 9-month females and when grouping both sexes. These results suggest that the structural changes are not the result of aging but rather of the influence of female sex hormones. The estrous cycle of female mice usually starts between the 6th – 8th week (White, 2007), but can be conditioned for many factors (Drickamer, 1974, 1984; Kruczek and Gruca, 1990), and prolongs itself to 14 months-old, when the animals are no longer reproductive (Flurkey et al., 2007). Furthermore, the variations we find in females, but not in males, when studying O-LM dendritic spine density are in accordance with previous findings from our laboratory studying these interneurons in intact and ovariectomized female GIN mice. We demonstrated that this parameter changes depending on the estrous phase in which the mouse is in, and the alterations seen in ovariectomized mice were also restored by 17β-Estradiol administration (Pérez-Rando et al., 2021). These structural changes had only been previously studied in pyramidal neurons (Woolley and McEwen, 1994; Luine and Frankfurt, 2013).

Interestingly, some of the effects of estrogens on hippocampal structure/connectivity appear to be mediated by NMDAR (El-Bakri et al., 2004). However, a more recent study revealed no differences in the mRNA levels of GluN1 and GluN2B in the dorsal hippocampus when comparing control and ovariectomized mice (McCarthny et al., 2018). Particularly, the study of NMDARs and hormonal levels during senescence demonstrated that NMDAR subunit mRNA levels in the hippocampus were much more prominently affected by the chronological age than by the reproductive status of the animals (Adams et al., 2001). In the present work we did not control the estrous cycle of the females, which could probably affect some parameters and constitute one of the limitations of the study.

Although we have not studied the density of SST-positive neurons, different studies have previously demonstrated a loss during aging in the hippocampus of rats, particularly in the *stratum oriens* (Potier et al., 2006; Stanley et al., 2012), although some reports did not find decreases in this cell type (Miettinen et al., 1993). Studies in mice are scarcer: one study described detrimental effects of aging on the number of these cells in the dentate gyrus (Koh et al., 2014), while another, using GIN mice, found an age-dependent decrease of O-LM interneurons from 5 to 9 months, but not from 9 to 12 months (Schmid et al., 2016). It is possible that these reductions of cell density may have an impact on the influence of SST-expressing interneurons during aging, in addition to the structural alterations that we have observed.

Interestingly, different studies have shown that aging is associated with a decrease in the excitability of hippocampal pyramidal neurons and with alterations in synaptic plasticity (LTP and LTD), which may underlie the deficits in hippocampal-dependent learning tasks (Oh and Disterhoft, 2020). These changes may be compensatory for a decrease in inhibition, because in the CA1 aging is associated with decreased GABAergic transmission (Billard et al., 1995; Potier et al., 2006), decreased number of GAD-67 + interneurons (Shetty and Turner, 1998; Stanley and Shetty, 2004), and decreased expression of GAD-67 mRNA in the CA1 (Vela et al., 2003). Specifically, the selective loss of O-LM cells in an age-dependent manner has been related to a decrease in the amplitude of evoked inhibitory postsynaptic currents (IPSCs) and a decrease in the frequency of spontaneous IPSCs in CA1 pyramidal neurons (Potier et al., 2006).

Previous studies in our laboratory have demonstrated that the structure of hippocampal SST-expressing interneurons in the *stratum oriens* of CA1 is regulated by NMDAR. Interestingly, we found that these glutamatergic receptors were expressed in the somata and dendritic spines of SST-expressing interneurons (Pérez-Rando et al., 2017a). In our previous studies using organotypic cultures, we observed that the relative density of spines and their appearance rate increased 24 h after the NMDA infusion (Pérez-Rando et al., 2017a). By contrast, the application of an NMDAR antagonist produced opposite results (Pérez-Rando et al., 2017b).

Mounting evidence suggests that an age-associated hypofunction of the NMDAR can contribute to the impairment in spatial learning and memory, particularly affecting the Schaffer collateral pathway (Foster and Norris, 1997; Kumar and Foster, 2013). A decrease in the expression of NMDARs has been observed in the hippocampus during normal aging (Magnusson et al., 2006; Billard and Rouaud, 2007; Zhao et al., 2009), particularly in the CA1 region (Magnusson and Cotman, 1993; Wenk and Barnes, 2000). However, the response to aging was different depending on the NMDAR subunit studied (Avila et al., 2017). There are no significant changes in GluN1 protein expression in the whole hippocampus of aged mice (Zhao et al., 2009) and particularly in the expression of GluN1 mRNA in the CA1 pyramidal layer (Magnusson et al., 2005). In the present report we have studied the density of GluN1 + puncta in the O-LM cells, but we did not observe changes during aging, which is in accordance with the previous data. However, decreases in the expression of GluN1 protein (Eckles-Smith et al., 2000; Mesches et al., 2004; Liu et al., 2008) and mRNA (Adams et al., 2001) in the whole hippocampus have been reported in aged rats. By contrast with GluN1, there is a widespread agreement that the GluN2B subunit is especially affected by aging. There is a decrease of its protein (Clayton and Browning, 2001; Mesches et al., 2004; Zhao et al., 2009) and mRNA expression (Adams et al., 2001; Clayton and Browning, 2001) in the whole hippocampus, and particularly in the CA1 pyramidal layer (Magnusson, 2001), both in rats and mice. It has to be noted, however, that these studies analyzed the whole hippocampus and not specific cell types as our experiment. Notwithstanding, in concordance with these previous studies, we observed a decrease of the GluN2B + puncta in the O-LM cells in our oldest group, which is significant in females and when analyzing both sexes together. However, the area covered by GluN2B + puncta in O-LM cells is considerably larger in the oldest animals, suggesting the presence of clusters of these receptors. In fact, it has been suggested that an increased association of GluN2B receptors with scaffolding proteins in aged animals may contribute to the age-related memory impairment (Zamzow et al., 2013). Additionally, there is data supporting that the age-associated hypofunction of NMDAR may be due to decrease in the activity-dependent changes in the surface distribution of NMDAR (Clayton et al., 2002) increased association with membrane scaffolding proteins (Zamzow et al., 2013) and to decreased trafficking of GluN2B to the synapse (Kumar et al., 2019). The clustering of GluN2B subunits may be the physical manifestation of poor trafficking of NMDAR, which possibly begins in middle-age.

It is interesting to note that the genetic deletion of GluN2B in interneurons prevents the formation of glutamatergic synapses on hippocampal interneurons (Kelsch et al., 2014). Consequently, it is possible that the age-related decrease in GluN2B expression in O-LM cells also interferes with the maintenance of their glutamatergic input, affecting thus the density of dendritic spines on these cells. Our results show that alterations in NMDAR do not only affect pyramidal neurons, but also SST-expressing cells and, consequently, the inhibition that these interneurons exert on excitatory cells. In fact, SST has a potent effect on LTP in CA1 (Rostampour et al., 2002; Fan and Fu, 2014).

An important limitation of our study is the age of the oldest animals (16 months). These mice are not considered aged in most studies, although cognitive impairment starts to appear around this age in rodents (Kumar and Foster, 2013). However, in our hands, most animals of this transgenic strain died shortly after this age and many of them presented tumors and considerable hair loss. Unfortunately, we were not able to perform learning tests in these animals and there are no reports analyzing cognitive tasks in aged animals of this strain or from this genetic background (FVB).

Altogether, our results help to shed light on how aging and sex modulate the structural plasticity and the NMDARs expression of hippocampal interneurons, particularly of O-LM cells. The study of these receptors is important since changes on their expression can lead to neuronal potentiation or depression, and alterations in their physiology may underlie behavioral and cognitive dysfunctions.

## Data Availability Statement

The raw data supporting the conclusions of this article will be made available by the authors, without undue reservation.

## Ethics Statement

The animal study was reviewed and approved by Committee on Bioethics of the Universitat de València.

## Author Contributions

JN designed the experiment. YG, PK, SC, and MP-R performed the experiments and quantifications. YG, PK, and JN wrote the manuscript and all the authors revised and edited the final version. All authors contributed to the article and approved the submitted version.

## Conflict of Interest

The authors declare that the research was conducted in the absence of any commercial or financial relationships that could be construed as a potential conflict of interest.

## Publisher’s Note

All claims expressed in this article are solely those of the authors and do not necessarily represent those of their affiliated organizations, or those of the publisher, the editors and the reviewers. Any product that may be evaluated in this article, or claim that may be made by its manufacturer, is not guaranteed or endorsed by the publisher.
